# Novel Multicomponent Titanate-Germanate Glasses: Synthesis, Structure, Properties, Transition Metal, and Rare Earth Doping

**DOI:** 10.3390/ma13194422

**Published:** 2020-10-04

**Authors:** Wojciech A. Pisarski, Karolina Kowalska, Marta Kuwik, Justyna Polak, Ewa Pietrasik, Tomasz Goryczka, Joanna Pisarska

**Affiliations:** 1 Institute of Chemistry, University of Silesia, Szkolna 9 Street, 40-007 Katowice, Poland; k.kowalska119@gmail.com (K.K.); marta.kuwik88@gmail.com (M.K.); justyna.polak@us.edu.pl (J.P.); ewa.pietrasik@us.edu.pl (E.P.); joanna.pisarska@us.edu.pl (J.P.); 2 Institute of Materials Science, University of Silesia, 75 Pułku Piechoty 1A Street, 41-500 Chorzów, Poland; tomasz.goryczka@us.edu.pl

**Keywords:** glasses, structure-property relationship, Cr^3+^, Eu^3+^, spectroscopic parameters

## Abstract

Novel multicomponent titanate-germanate glasses singly doped with transition metal (Cr^3+^) and rare earth ions (Eu^3+^) were synthesized and the glass transition temperatures and thermal stability parameters were determined using DSC measurements. X-ray diffraction analysis confirmed fully amorphous nature of the received samples. Their structural and optical properties were compared with germanate glasses without TiO_2_. Correlation between local structure and optical properties in titanate-germanate glasses is well evidenced by FT-IR, Raman, EPR, and luminescence spectroscopy. In particular, luminescence spectra and their decays are examined for glass samples, where GeO_2_ was partially substituted by TiO_2_.

## 1. Introduction

Since 1986 the formation of TiO_2_ containing glasses has been investigated in detail [1,2,3,4,5,6,7,8]. Unfortunately, most of titanate glass systems are partly crystallized. The obtained systems possess crystalline phases mainly because of different titanates and their thermal stability parameters are relatively low, which makes them unsuitable for optical-fiber applications. In fact, it is difficult to prepare thermally stable and fully amorphous systems with relatively high titanium oxide content. On the other hand, germanate glasses have quite strong chemical and mechanical stability useful for optical fiber drawing and belong to low-phonon glass family. Compared to other low-phonon glass systems such as tellurite glasses, germanate based glass-host matrices have relatively large glass-forming region. In particular, thermal stability parameter referred to as a difference between crystallization onset T_x_ and glass transition temperature T_g_ is considerably higher for germanate-based glass with ΔT = 155 °C [9] than tellurite based glass with ΔT = 27 °C [10]. Quantum efficiencies for ^4^F_3/2_ → ^4^I_11/2_ (Nd^3+^) and ^4^I_13/2_ → ^4^I_15/2_ (Er^3+^) transitions of rare earth ions in germanate glasses based on GeO_2_-BaO-Ga_2_O_3_ are close to 80% [11] and 71% [12], respectively. Their values are also larger compared to main near-infrared laser transitions of Nd^3+^ (η = 68%) and Er^3+^ (η = 46%) ions in glasses based on TeO_2_-ZnO [13,14]. Various glass-modifiers were tested in order to obtain thermally stable and amorphous systems with excellent luminescence properties. Systematic studies clearly indicate that the effect of modifier oxides on emission properties of rare earth ions in different glass matrices is significant [15]. Influence of modifier oxides M_2_O where M denotes Li, Na, K, Rb, Cs [16], MO where M = Ca, Sr, Ba [17,18], M_2_O_3_ where M = Al or Ga [19] and MO_2_ where M = Te, Ge, Si [20] on local structure of glasses and their multifunctional properties and potential applications has been presented and discussed. Special attention has been paid to germanate glasses with different glass-modifiers. High niobium oxide content in alkali germanate glasses was evidenced by the optical absorption, DSC and XRD analysis, FT-IR, and Raman spectroscopy. Marcondes et al. [21] suggest that high niobium oxide content causes an increase in the glass-host network and strongly modifies thermal, structural, and optical properties of alkali germanate glasses. These structural and optical aspects for Eu^3+^ doped germanate glasses modified by MO/MF_2_ where M denotes Ca, Sr, Ba, have been also studied [22]. In particular, the influence of the oxide and fluoride glass-modifiers on local structure of germanate glasses has been examined using X-ray diffraction analysis. The experimental results clearly demonstrate that samples with modifiers MO/MF_2_ (M = Ca or Sr) are crystalline, whereas samples with BaO and/or BaF_2_ are fully amorphous. Further studies revealed that modification of germanate glasses by P_2_O_5_ allows control of their local structure and visible luminescence. The increase of P_2_O_5_ content leads to the reduction of spectral linewidth and the shift of emission band of Eu^3+^ ions in germanate glass to shorter wavelengths [23]. The effect of the spectroscopic properties of Tm^3+^ ions for different compositions with varying Nb_2_O_5_/La_2_O_3_ ratios has been studied and the optical concentration of glass components for efficient 1.8 µm near-infrared laser applications was determined [24]. Rare earth-doped germanate glasses modified by Bi_2_O_3_ [25], Y_2_O_3_ and Nb_2_O_5_ [26] have been also analyzed for mid-infrared emission. These aspects were not yet examined for germanate-based glass in the presence of titanium dioxide.

In the present work, multicomponent glasses based on TiO_2_-GeO_2_-BaO-Ga_2_O_3_-M_2_O_3_ (M—rare earth or transition metal) were successfully synthesized using conventional high-temperature melting and their structure and properties are presented and compared to the glasses in the absence of TiO_2_. Local structure and properties of multicomponent glasses containing two glass-network formers GeO_2_ and TiO_2_ were characterized using various experimental techniques: X-ray diffraction (XRD), differential scanning calorimetry (DSC), electron paramagnetic resonance (EPR), Raman and Fourier-transform infrared spectroscopy (FT-IR), absorption and luminescence spectroscopy. Transition metal (Cr^3+^) and rare earth (Eu^3+^), commonly known as spectroscopic probe, were used as the optical dopants. Our new preliminary results for titanate-germanate glasses are presented and discussed in relation to potential visible (Eu^3+^) and near-infrared (Cr^3+^) luminescence applications. In particular, luminescence spectra and decay curves were examined for glass samples, where germanium dioxide was substituted by titanium dioxide and the relative molar ratio of these two main glass-former components is equal to GeO_2_:TiO_2_ = 1:1. In previous work TiO_2_ was substituted by GeO_2_ in multicomponent germanoniobophosphate glass system allowing the glass stabilization against devitrification and the improvement of photoluminescence behavior, but amount of titanium dioxide playing the role as glass-network modifier did not exceed 15 molar % [27].

## 2. Materials and Methods 

Multicomponent glasses undoped and doped with transition metal or rare earth were prepared: 30TiO_2_-30GeO_2_-30BaO-10Ga_2_O_3_ (referred as TiGe), 30TiO_2_-30GeO_2_-30BaO-9.75Ga_2_O_3_-0.25Cr_2_O_3_ (TiGe-Cr), 30TiO_2_-30GeO_2_-30BaO-9.75Ga_2_O_3_-0.5Eu_2_O_3_ (TiGe-Eu) and their structure and properties were compared to glass samples without titanium dioxide 60GeO_2_-30BaO-10Ga_2_O_3_ (referred as Ge), 60GeO_2_-30BaO-9.75Ga_2_O_3_-0.25Cr_2_O_3_ (Ge-Cr), and 60GeO_2_-30BaO-9.75Ga_2_O_3_-0.5Eu_2_O_3_ (Ge-Eu). The concentrations of components are given in molar %. Titanate-germanate glasses were synthesized using high-temperature melt quenching-technique. The appropriate amounts of glass components (metal oxides of high purity 99.99%, Aldrich Chemical Co., St. Louis, MO, USA) were mixed and melted (1200 °C/0.45 h).

The amorphous nature of samples was confirmed by X-ray diffraction measurements (X’Pert Pro diffractometer, Panalytical, Almelo, The Netherlands) with Cu K_α1_ radiation (λ = 1.54056 Å). The Cu X-ray tube operating at 40 kW/30 mA was used. Diffraction patterns were measured in step-scan mode with a step size of 0.05^0^ and time per step of 10 s. The glass samples were characterized by a SETARAM Labsys thermal analyzer (SETARAM Instrumentation, Caluire, France) using the DSC method. The DSC curves were acquired with heating rate of 10 °C /min.

The electron paramagnetic resonance spectra were performed using Bruker EMX EPR spectrometer (Bruker-Biospin, Karlsruhe, Germany) working at X-band frequency (9.8 GHz). The EPR instrument parameters are as follows: central field 3480 G, modulation amplitude 2.0 G, time constant 40.96, gain 1 × 10^4^ G, and microwave power 20.12 mW. The infrared spectra using the ATR technique were recorded over the frequency range of 1000–350 cm^−^^1^ using a Nicolet™ iS™ 50 FT-IR spectrometer (Thermo Fisher Scientific, Waltham, MA, USA) with a diamond attenuated total reflectance (ATR) module. The Raman spectra using a Thermo Fisher Scientific™ DXR™2xi Raman Imaging Microscope and laser working as the source (24 mW power) with excitation wavelength 780 nm were measured. The laser was directly focused on the glass sample with an Olympus long-working-distance microscope objective (50×).

Next, the glass samples were characterized using absorption (Varian Cary 5000 UV-VIS-NIR spectrophotometer, Agilent Technology, Santa Clara, CA, USA) and luminescence spectroscopy (laser equipment, which consists of PTI QuantaMaster QM40 spectrofluorometer, tunable pulsed optical parametric oscillator (OPO), Nd:YAG laser (Opotek Opolette 355 LD, Carlsbad, CA, USA), double 200 mm monochromators, multimode UVVIS PMT R928 and Hamamatsu H10330B-75 detectors (Hamamatasu, Bridgewater, NJ, USA), PTI and ASOC-10 USB-2500 oscilloscope). Resolution for spectral measurements was ±0.1 nm, whereas decay curves with accuracy ±0.5 µs were acquired.

## 3. Results and Discussion

### 3.1. Undoped Titanate-Germanate Glasses 

**Figure 1:**
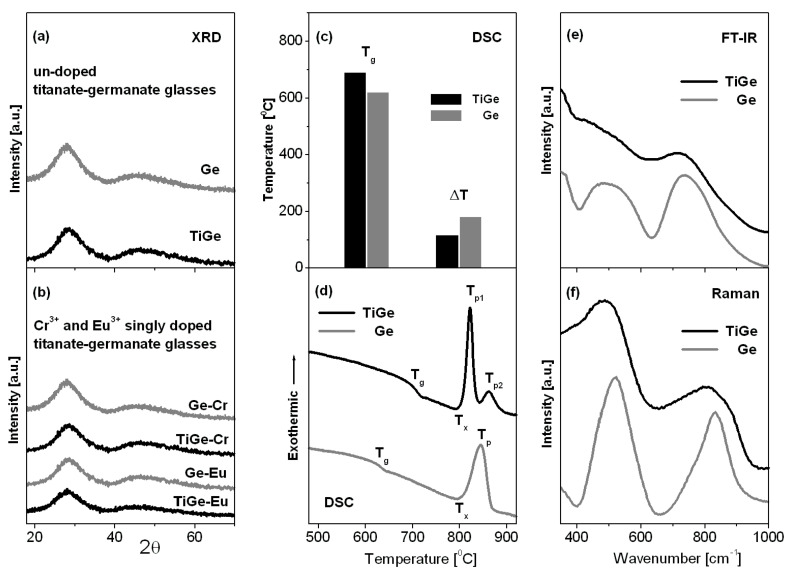
X-ray diffraction patterns (**a**,**b**), thermal parameters T_g_ and ΔT (**c**) and DSC curves (**d**), FT-IR (**e**) and Raman (**f**) spectra measured for titanate-germanate glasses (TiGe) and compared to glass samples without TiO_2_ (Ge).

Titanate-germanate glasses were successfully synthesized and their structure and properties were examined using XRD, DSC, FT-IR, and Raman spectroscopy. Figure 1 shows XRD patterns (a, b), DSC curves (c, d), FT-IR (e), and Raman (f) spectra for titanate-germanate glasses referred as TiGe. They are compared to the results obtained for glass samples without titanium dioxide (Ge).

The received glass samples (TiGe and Ge) reveal X-ray diffraction patterns characteristic for amorphous systems and narrow diffraction lines typical for crystalline materials are not observed. Moreover, any significant structural changes in the XRD patterns have been observed for glass samples after transition metal (TiGe-Cr) or rare earth (TiGe-Eu) doping. It clearly indicates that titanate-germanate glasses are able to accommodate transition metal or rare earth ions and the samples are still fully amorphous. Our previous studies for lead borate glasses demonstrated that rare earth oxides influence on the resistance to crystallization. In contrast to sample with Nd_2_O_3_, several crystalline peaks due to the ErBO_3_ phase are present after addition of Er_2_O_3_ to the base lead borate glass, suggesting the increased tendency toward crystallization [28]. From DSC curves measured for glass samples (TiGe and Ge), the glass transition temperature T_g_ and thermal stability parameter (ΔT = T_x_ − T_g_) were determined. In contrast to germanate glass (Ge), the additional exothermic peak representing the crystallization of the glass can be observed for glass sample with the presence of TiO_2_. It is well evidenced that the thermal stability parameter is reduced where GeO_2_ is partially replaced by TiO_2_. The glass transition temperature T_g_ increases from 620 °C to 690 °C suggesting less open glass structure [29]. These thermal parameters T_g_ and ΔT are also schematized on Figure 1c. The Raman and FT-IR spectra between 350 cm^−1^ and 1000 cm^−1^ frequency region consists of two main bands centered at about 500 cm^−1^ and 800 cm^−1^. Similar to previous reports for germanate-based glasses [30,31], the low-frequency band located from 400 cm^−1^ to 600 cm^−1^ is assigned to bending vibration involving Ge-O-Ge and Ge-O-Ga bridges, whereas the high-frequency band between 700 cm^−1^ and 900 cm^−1^ is attributed to asymmetric stretching vibrations of Ge-O-Ge bonds and symmetric stretching of Ge-O/Ga-O bonds. In general, Raman and FT-IR bands are shifted to lower frequency region in the presence of TiO_2_. Kamitsos et al. [31] observed similar effects for germanate glasses in function of Rb_2_O. In the 630–700 cm^−1^ frequency region, the additional band located near 650 cm^−1^ is quite well observed for glass sample with titanium dioxide. This band is due to the stretching vibration of Ti-O in TiO_6_ unit [32].

### 3.2. Titanate-Germanate Glasses Doped with Chromium Ions

**Figure 2:**
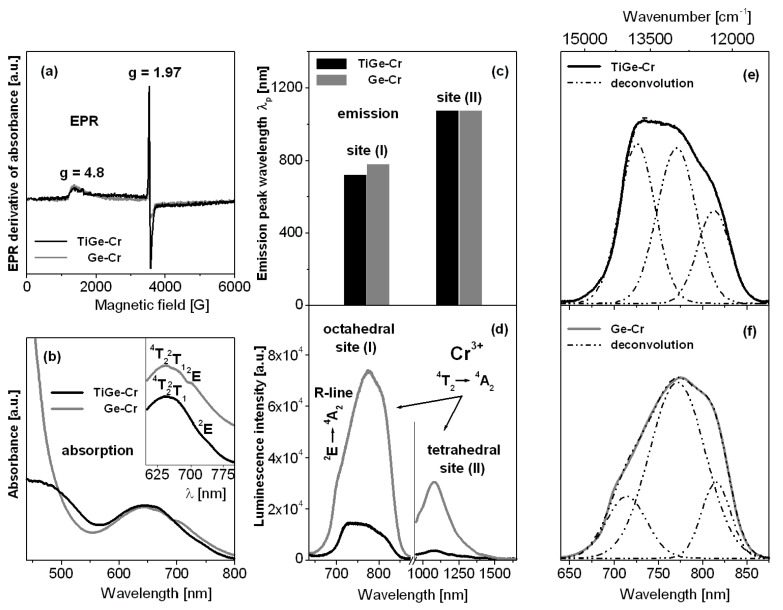
EPR (**a**), absorption (**b**), and luminescence (**c**,**d**) spectra measured for titanate-germanate glasses doped with chromium ions (TiGe-Cr) and compared to glass samples without TiO_2_ (Ge-Cr). Deconvoluted emission bands for both glass samples TiGe-Cr (**e**) and Ge-Cr (**f**) are also given.

Figure 2 shows results for titanate-germanate glasses doped with chromium ions, which were characterized using EPR (a), absorption (b), and luminescence (c–f) spectroscopy. Independently on samples with the presence (TiGe-Cr) or absence (Ge-Cr) of TiO_2_, the EPR spectra show two resonant signals at about g = 4.8 and g = 1.97, which evidently proves the 3+ valence state for chromium ions and the octahedral coordination. The similar effects were observed earlier for trivalent chromium ions in lead niobium germanosilicate glasses [33] and antimony phosphate glasses [34]. These two resonance signals may be quite well interpreted. They are related to the isolated Cr^3+^ ions (g = 4.8) and the exchange coupled pairs Cr^3+^-Cr^3+^ (g = 1.97) [35]. The presence of chromium ions at trivalent state in the studied glass systems was also confirmed by the absorption spectra measurements. The spectra measured in 550–800 nm ranges show characteristic broad absorption band, which consist of three overlapped peaks due to transitions originating from ^4^A_2_ ground state to the ^4^T_2_, ^2^T_1_, and ^2^E excited states of trivalent chromium, respectively. Comparing to sample Ge-Cr, the ^4^A_2_ → ^2^E transition of Cr^3+^ ions is shifted to longer wavelengths in the presence of titanium dioxide (TiGe-Cr). At this moment, it should be also noticed that the second absorption band associated to the ^4^A_2_ → ^4^T_1_ transition of Cr^3+^ ions is located at about 430 nm. This band has not been observed for several glasses, because it is masked by strong UV-vis absorption of the host or lies on the tail of absorption edge. However, both absorption bands of chromium ions were successfully measured by us for barium gallo-germanate glass. Thus, some important spectroscopic parameters were calculated owing to the Tanabe-Sugano diagram for d^3^ electronic configuration suggesting that chromium ions in barium gallo-germanate glass are in an intermediate octahedral ligand field environment (2.1 < Dq/B < 2.3). The crystal field parameters, the Racah parameters, and the related ligand field parameters are as follows: Dq = 1557 cm^−1^, B = 732 cm^−1^, C = 2991 cm^−1^, and Dq/B = 2.13 [36]. 

The near-infrared luminescence spectra of chromium ions revealed two emission bands, but both well observed lines are assigned to the transition originating from the ^4^T_2_ excited state to the ^4^A_2_ ground state. According to the excellent paper published recently, the near-infrared emission bands centered at about 730 nm and 1030 nm are related to the ^4^T_2_ → ^4^A_2_ transitions in octahedral sites (I) and tetrahedral sites (II) of chromium ions [37]. Further spectroscopic analysis indicates that the intensities of luminescence bands are stronger for chromium ions located at octahedral site (I) than tetrahedral site (II). The maximum of emission peak wavelength for the ^4^T_2_ → ^4^A_2_ transition of chromium ions in octahedral sites (I) is changed from 730 nm (Ge-Cr) to 775 nm in the presence of titanium dioxide (TiGe-Cr) in contrast to tetrahedral site (II), where both peak maxima are the same. Furthermore, the profiles of emission bands associated to transition of chromium ions in site (I) are completely different. It is especially evidenced for the ^2^E → ^4^A_2_ transition commonly known as R-line, which is overlapped with the ^4^T_2_ → ^4^A_2_ transition of chromium ions in octahedral site (I). The maximum of R-line is shifted to longer wavelength from 715 nm (Ge-Cr) to 727 nm (TiGe-Cr). In order to further study the structural changes occurring in the arrangement around Cr^3+^, the emission bands were successfully deconvoluted into three Gaussian components. The luminescence band ascribed to the ^4^T_2_ → ^4^A_2_ transition was well divided into the red and the blue components, confirming the coexistence of two completely different site distributions for chromium ions. During the deconvolution procedure, peak wavenumber (ν), linewidth (dν), the energy gap between both ^4^T_2_ and ^2^E excited states ΔE = E(^2^E) − E(^4^T_2_), and the relative integrated emission line intensities *I*(^2^E)/*I*(^4^T_2_) = A_R-LINE_/(A_RED_ + A_BLUE_) and *I*(^2^E)/*I*_TOTAL_) = A_R-LINE_/(A_RED_ + A_BLUE_ +A_R-LINE_) were estimated. The A_RED_ + A_BLUE_ +A_R-LINE_ denotes the integrated emission intensities of the red and the blue components of ^4^T_2_ as well as ^2^E (*R*-line), respectively. The relative integrated intensities of the bands were measured in order to monitor the equilibrium position between the ^4^T_2_ and ^2^E excite states of chromium. The results are summarized in Table 1.

Our calculations give interesting results. The energy gap between the ^4^T_2_ and ^2^E excited states of chromium ions increases significantly from 792 cm^−1^ (TiGe-Cr) to 1005 cm^−1^ in the glass sample with the absence of titanium dioxide (Ge-Cr). The relative integrated emission line intensities denoted as *A*_RED_, *A*_BLUE_, and *A*_R-LINE_ due to the ^4^T_2_ → ^4^A_2_ and ^2^E → ^4^A_2_ transitions of chromium ions are also drastically changed. Thus, the relative integrated line intensity ratios of *I*(^2^E)/*I*(^4^T_2_) and *I*(^2^E)/*I*_TOTAL_) are increased with the presence of TiO_2_ in glass composition. The appropriate relative integrated line intensity ratios increase from 0.21 to 0.63 (*I*(^2^E)/*I*(^4^T_2_)) and from 0.17 to 0.39 (*I*(^2^E)/*I*_TOTAL_)), when GeO_2_ was partially substituted by TiO_2_, respectively. It suggests that chromium ions occupy higher crystal-field sites in germanate glasses in the presence of TiO_2_. Completely different situation was observed previously for chromium ions in lead borate glass. The energy gap between ^4^T_2_ and ^2^E states was changed from 1055 cm^−1^ (PbO:B_2_O_3_ = 1:1) to 770 cm^−1^ (PbO:B_2_O_3_ = 4:1). In this case, the same Gaussian-fitting procedure was applied to evaluate spectroscopic parameters. The relative integrated line intensity ratios were nearly twice reduced, suggesting the presence of chromium ions in lower crystal-field sites with increasing PbO concentration [38].

Spectroscopic results for the studied glasses suggest that photoluminescence properties of chromium ions depend critically on titanium dioxide. For germanate glass in presence of TiO_2_ (TiGe-Cr), the Cr^3+^ ions are located in the higher crystal field and, thus, the emission of sharp R-line arising from the spin-forbidden ^2^E → ^4^A_2_ transition is more intense. When the Cr^3+^ ions are located in the lower crystal field, broadband emission originating from the spin-allowed ^4^T_2_ → ^4^A_2_ transition is dominated (Ge-Cr). There is in a good agreement with the results obtained previously for fluoride-sulfophosphate glasses, which are promising hosts for broadband optical amplification through transition metal activators [39].

### 3.3. Titanate-Germanate Glasses Doped with Europium Ions

**Figure 3:**
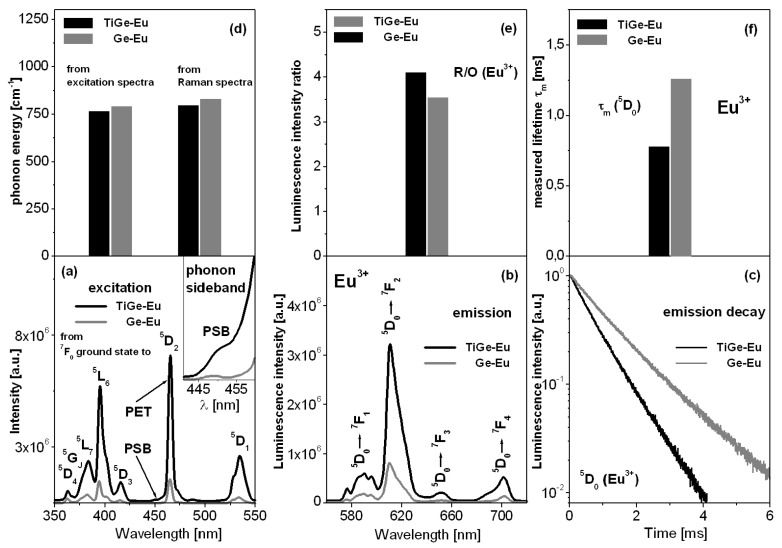
Excitation (**a**) and emission (**b**) spectra, and decay curves (**c**) for titanate-germanate glasses doped with europium ions (TiGe-Eu) and compared to glass samples without TiO_2_ (Ge-Eu). All changes are also schematized (**d**–**f**).

Excitation (Figure 3a) and emission (Figure 3b) spectra, and decay curves (Figure 3c) measured for titanate-germanate glasses doped with europium ions (TiGe-Eu) are presented in Figure 3. The results are compared to glass samples without TiO_2_ (Ge-Eu). All changes are also schematized on Figure 3d–f.

The excitation spectrum consists of several bands, which originate from the ^7^F_0_ ground state to the higher-lying ^5^D_2_, ^5^D_3_, ^5^L_6_, ^5^L_7_, ^5^G_J_, and ^5^D_4_ excited states of europium ions. The most intense bands are due to ^7^F_0_ → ^5^L_6_ (near 390 nm) and ^7^F_0_ → ^5^D_2_ (near 460 nm) transitions. The later transition is known as the pure electronic transition (PET). In this spectral region, the phonon sideband (PSB) is also located and associated with the pure electronic transition (PET). The difference between the positions of both PSB and PET bands is well-known as the phonon energy of the host. Our studies indicate that the phonon energy of the glass host is reduced from 790 cm^−1^ (Ge-Eu) to 765 cm^−1^ with the presence of TiO_2_ (TiGe-Eu). From phonon sideband measurements [40,41,42], the electron–phonon coupling strength g can be also estimated, which is due to the intensity ratio of the PSB (∫I_PSB_ dν) to the PET (∫I_PET_ dν), respectively. The results are given in Table 2.

Finally, the multiphonon relaxation rate Wp(T) depending on the electron–phonon coupling strength and phonon energy of the glass host can be determined as follows W_p_(T) = W_0_(0)exp(−αΔE), where W_0_(0) is the transition probability extrapolated to zero energy gap, ΔE denotes the energy gap between neighboring energy states and the values of ^5^D_1_–^5^D_0_ and ^5^D_2_–^5^D_1_ energy gaps of Eu^3+^ ions are equal nearly to 1750 cm^−1^ and 2500 cm^−1^, respectively. In this relation the α parameter is close to (ln(p/g)−1)/hω, where hω represents the phonon energy, g—the electron-phonon coupling strength, and p as the phonon number is equal to ΔE/hω. In some cases, the multiphonon relaxation rate is given as W_p_(T)/W_0_(0) [43]. Our spectroscopic calculations presented in Table 2 clearly indicate that the electron-phonon coupling strength and multiphonon relaxation rates from the ^5^D_1_ and ^5^D_2_ states of europium ions are significantly smaller for glass sample with the presence of titanium dioxide.

Further experimental investigations shown on Figure 3 suggest that titanate-germanate glass demonstrates the efficient reddish-orange emission independently on the excitation wavelengths at 390 nm (^5^L_6_ state) or 460 nm (^5^D_2_ state) and its intensity is considerably higher in comparison to the glass sample without titanium dioxide. The emission bands correspond to the electronic transitions originating from the ^5^D_0_ state to the ^7^F_J_ (J = 1–4) states of europium ions, respectively.

In order to evaluate the glass asymmetry and the strength of bonding (covalent/ionic character) between europium ions and their surroundings, the ratio of integrated band intensity of ^5^D_0_ → ^7^F_2_ transition to that of the ^5^D_0_ → ^7^F_1_ transition, well-known in literature as red-to-orange factor R/O (Eu^3+^), was calculated. It is generally accepted that the value of R/O (Eu^3+^) starts to increase with increasing local asymmetry and covalent bonding. This phenomenon is just observed for our glass sample with the presence of titanium dioxide. For the studied systems, the fluorescence intensity ratio R/O (Eu^3+^) was changed from 3.54 (Ge-Eu) to 4.10 (TiGe-Eu).

Based on decay curve measurements, the luminescence lifetimes for the ^5^D_0_ state of europium ions were also determined. In general, the multiphonon relaxation rates decrease with decreasing phonon energy of the glass-host and consequently the lifetimes measured for excited states of rare earths are usually enhanced. Completely opposite situation is observed for europium ions, because the energy gap between ^5^D_0_ state and lower-lying ^7^F_6_ state of europium ions is very large. Its value seems to be nearly 12,500 cm^−1^. In this case, several phonons are needed to bridge energy gap and radiative relaxation is a dominant transition. For low-phonon glass systems containing europium ions, the non-radiative relaxation rate is negligibly small in contrast to the radiative relaxation rate. Thus, the nonradiative relaxation rate can be ignored. In a practice, the radiative relaxation rate represents total relaxation rate. The spectroscopic consequence is reduction of luminescence lifetime (as an inverse of total radiative relaxation rate) for the ^5^D_0_ state of europium ions with decreasing phonon energy of the glass-host. These phenomena were presented and discussed in our previous published work [44]. The analysis of luminescence decay curves for glass samples with the presence and absence of titanium dioxide confirms the hypothesis given above. The measured lifetime τ_m_ for the ^5^D_0_ state of europium ions is reduced from 1.26 ms (Ge-Eu) to 0.78 ms (TiGe-Eu), when the phonon energy decreases from 790 cm^−1^ to 765 cm^−1^ for glass sample with the presence of TiO_2_. It suggests that the influence of titanium dioxide on spectroscopic parameters of europium ions is significant. The changes of phonon energy, fluorescence intensity ratio R/O, and ^5^D_0_ measured lifetime of Eu^3+^ ions in function of TiO_2_ are also schematized in Figure 3d–f.

The effects of TiO_2_ on luminescence behavior of germanate glass depend greatly on the kind of active dopants (transition metal or rare earth). Luminescence properties of germanate glasses in the presence of TiO_2_ are completely different for europium ions than chromium ions discussed in previous Part 3.2. In particular, the intensities of luminescence bands of the optically active ions are changed drastically, when GeO_2_ was substituted by TiO_2_ in the glass composition. The intensities of luminescence bands of chromium ions are reduced, whereas the emission band intensities of europium ions increase significantly in the presence of TiO_2_. Compared to the results for similar glass-hosts published recently [45,46,47,48], we postulate that our multicomponent titanate-germanate glass doped with Eu^3+^ ions is suitable as red-emitting component for LED applications.

First of all, the significant advantage of our systems is the lower phonon energy of the host in comparison to other oxide glasses such as borate (~1400 cm^−1^), phosphate (~1200 cm^−1^), and silicate (~1050 cm^−1^) systems [49,50,51]. It is assumed that glasses with low phonon energy are more suitable as host materials for rare earth ions because of less probable non-radiative relaxation process, which may result in a higher lifetime of the excited state. According to experimental results presented in Table 3, the luminescence lifetime for ^5^D_0_ excited state evaluated for glass samples (Ge-Eu and TiGe-Eu) is similar to the values obtained for various germanate systems doped with europium ions [52,53,54,55,56,57,58,59,60]. However, as has already been discussed for low-phonon glass-hosts containing Eu^3+^ ions, the non-radiative relaxation process can be neglected. Thus, the luminescence lifetime as an inverse of total radiative relaxation rate reduces with decreasing phonon energy of the glass-host. Therefore, the value of lifetime for ^5^D_0_ state in glass sample Ge-Eu (790 cm^−1^) is longer than the value of lifetime for systems with titanium oxide (765 cm^−1^), lead oxide (775 cm^−1^), and bismuth oxide (770 cm^−1^) [51,52,53]. Moreover, it was stated that the fluorescence intensity ratio R/O (Eu^3+^) was increased from 3.54 (Ge-Eu) to 4.10 (TiGe-Eu) for the glass samples and the value of this spectroscopic parameters is significantly higher than those of lead germanate glasses [52,57] antimony [55] and tellurium [56] oxides-modified germanate systems. The obtained value of the ratio R/O indicates the higher local asymmetry around the Eu^3+^ ions in the glass host. It is worth noting that this factor confirms that the addition of a high concentration of titanium dioxide to the germanate matrix does not prompt the crystallization process, although TiO_2_ can be a nucleating agent in glass host. Taking this issue into consideration, further research is needed to determine if a thermally stable and fully amorphous system with relatively high titanium oxide content is a good candidate for optical materials, that may find potential use in photonic devices such as optical fibers and amplifiers.

## 4. Conclusions

Multicomponent titanate-germanate glasses undoped and doped with transition metal (Cr^3+^) and rare earths (Eu^3+^) were prepared and then characterized using various experimental techniques: DSC, XRD, EPR, FT-IR, Raman, and luminescence spectroscopy. X-ray diffraction analysis revealed that all received samples are fully amorphous. Thermal and structural studies indicate that the glass transition temperature increases and thermal stability factor is reduced, whereas the Raman and FT-IR bands are shifted to lower frequency region in the presence of TiO_2_. The EPR spectra show typical signals confirming the presence of Cr^3+^ ions at trivalent state and the octahedral coordination. From the excitation spectra (phonon sideband analysis) of Eu^3+^, the phonon energy of the glass-host, the electron–phonon coupling strength, and the multiphonon relaxation rate were also determined.

In particular, luminescence spectra have been examined for glass samples, where germanium dioxide was substituted by titanium dioxide as well as the relative molar ratio of two glass-former components is equal to GeO_2_:TiO_2_ = 1:1. Near-infrared luminescence spectra of chromium ions show two emission bands near 730 nm and 1030 nm, which correspond to the ^4^T_2_ → ^4^A_2_ transitions in octahedral and tetrahedral sites, respectively. Further spectral analysis suggests that chromium ions occupy higher crystal-field sites in germanate glass with the presence of titanium dioxide. Visible luminescence spectra of europium ions present characteristic emission bands associated to ^5^D_0_ → ^7^F_J_ (J = 1–4) transitions. The red-to-orange fluorescence intensity ratio R/O and the luminescence lifetime for the ^5^D_0_ state of europium were determined. The later parameter, i.e., the measured ^5^D_0_ lifetime was reduced from 1.26 ms (Ge-Eu) to 0.78 ms (TiGe-Eu). This behavior is quite well correlated with the phonon energy, which decreases from 790 cm^−1^ to 765 cm^−1^ with the presence of titanium dioxide. The factor R/O was changed from 3.54 (Ge-Eu) to 4.10 (TiGe-Eu) suggesting the increase of local asymmetry and stronger covalent character of bonding between europium ions and their nearest surroundings in glass sample in the presence of TiO_2_. 

Our spectroscopic studies clearly indicate that luminescence properties of multicomponent titanate-germanate glasses are completely different for transition metal ions than rare earth ions. The intensities of emission bands of chromium ions are reduced, whereas the emission band intensities of europium ions increase drastically in the presence of TiO_2_. The obtained results demonstrate that titanate-germanate glass doped with Eu^3+^ ions is a promising candidate for red luminescence applications.

## Figures and Tables

**Table 1:** Spectroscopic parameters for chromium ions in glasses TiGe-Cr and Ge-Cr.

Spectroscopic Parameter	TiGe-Cr	Ge-Cr
ν_RED_ (^4^T_2_) (cm^−1^)	12,326	12,266
ν_BLUE_ (^4^T_2_) (cm^−1^)	12,963	12,981
ν_R-LINE_ (^2^E) (cm^−1^)	13,755	13,986
ΔE (cm^−1^)	792	1005
dν_RED_ (^4^T_2_) (cm^−1^)	518	480
dν_BLUE_ (^4^T_2_) (cm^−1^)	711	900
dν_R-LINE_ (^2^E) (cm^−1^)	838	1082
*I*(^2^E)/*I*(^4^T_2_)	0.63	0.21
*I*(^2^E)/*I*_TOTAL_)	0.39	0.17

**Table 2:** Spectroscopic parameters for europium ions in glasses TiGe-Eu and Ge-Eu.

Spectroscopic Parameter	TiGe-Eu	Ge-Eu
PSB–PET (cm^−1^)	765	790
Electron-phonon coupling strength g (×10^−3^)	1.7	5.2
Non-radiative relaxation rate W_p_(T)/W_0_(0) (s^−1^)		
from ^5^D_1_ state	6.85 × 10^−7^	1.37 × 10^−5^
from ^5^D_2_ state	4.87 × 10^−10^	3.66 × 10^−8^

**Table 3:** Comparison of spectroscopic parameters of Eu^3+^-doped germanate glasses.

Glass Composition	R/O	τ_m_ (ms)	References
Ge-Eu	3.54	1.26	present work
TiGe-Eu	4.10	0.78	present work
PbO-GeO_2_-Ga_2_O_3_	3.06	1.11	[52]
Bi_2_O_3_-GeO_2_	3.94	1.03	[53]
GeO_2_-Nb_2_O_5_-Li_2_O	6.50	0.81	[54]
GeO_2_-Nb_2_O_5_-Na_2_O	9.00	0.76	[54]
GeO_2_-Ga_2_O_3_-BaO-Sb_2_O_3_	1.94	-	[55]
GeO_2_-Ga_2_O_3_-BaO-TeO_2_	2.49	-	[56]
PbO-GeO_2_	2.86	-	[57]
Sb_2_O_3_-GeO_2_-B_2_O_3_-Al_2_O_3_-Na_2_O	1.80	-	[58]
GeO_2_-B_2_O_3_-Al_2_O_3_-Lu_2_O_3_-Gd_2_O_3_	-	1.43	[59]
GeO_2_-PbO	-	1.10	[60]
